# Management of periocular keratinocyte carcinomas with Mohs micrographic surgery and predictors of complex reconstruction: a retrospective study^☆^

**DOI:** 10.1016/j.abd.2023.05.004

**Published:** 2023-11-20

**Authors:** Dominga Peirano, Sebastián Vargas, Leonel Hidalgo, Francisca Donoso, Eugenia Abusleme, Felipe Sanhueza, Consuelo Cárdenas, Katherine Droppelmann, Juan Camilo Castro, Pablo Uribe, Pablo Zoroquiain, Cristian Navarrete-Dechent

**Affiliations:** a Department of Dermatology, Escuela de Medicina, Pontificia Universidad Católica de Chile, Santiago, Chile; b Department of Ophtalmology, Escuela de Medicina, Pontificia Universidad Católica de Chile, Santiago, Chile; c Melanoma and Skin Cancer Unit, Escuela de Medicina, Pontificia Universidad Católica de Chile, Santiago, Chile; d Department of Pathology, Escuela de Medicina, Pontificia Universidad Católica de Chile, Santiago, Chile

**Keywords:** Carcinoma, Eyelid neoplasms, Keratinocytes, Mohs surgery, Neoplasms, Skin

## Abstract

**Background:**

Skin cancer is the most frequent cancer worldwide and the most frequent periocular tumor. Keratinocyte Carcinomas (KC) located in periorificial areas, such as periocular tumors, are considered high-risk tumors. Mohs Micrographic Surgery (MMS) is considered the first line for the treatment of high-risk KC, providing a lower recurrence rate than conventional wide excision.

**Objective:**

To describe the clinical-pathological features of periocular KC treated with MMS in a tertiary university center in Chile.

**Methods:**

A single-center, retrospective study of patients with KC located on the periocular area, that underwent MMS between 2017‒2022. MMS details were recorded.

**Results:**

One hundred thirteen patients with periocular carcinomas were included. The mean age was 59 ± 13 years; 52% were women. The most frequent location was the medial canthus (53%), followed by the lower eyelid (30.1%). The most frequent BCC histology was the nodular variant (59.3%). Regarding MMS, the average number of stages was 1.5 ± 0.7, and 54% of the cases required only 1 stage to achieve clear margins. To date, no recurrence has been reported. Tumors larger than 8.5 mm in largest diameter or 43.5 mm^2^ were more likely to require complex reconstruction.

**Study limitations:**

Retrospective design and a relatively low number of patients in the SCC group. Possible selection bias, as larger or more complex cases, may have been referred to oculoplastic surgeons directly.

**Conclusion:**

The present study confirms the role of MMS for the treatment of periocular KCs. Periocular KCs larger than 8.5 mm might require complex reconstruction. These results can be used to counsel patients during pre-surgical visits.

## Introduction

Skin Cancer is the most frequent cancer worldwide and the most frequent periocular tumor.^1^, ^2^ About 5%–10% of all Keratinocyte Carcinomas (KC) occur in the periocular region and many are thought to be associated with chronic cumulative Ultraviolet (UV) light exposure.^3^, ^4^, ^5^ KC comprises Basal Cell Carcinoma (BCC) and Squamous Cell Carcinoma (SCC), being BCC the most frequent.^4^, ^6^ It's known that the prevalence of KC varies between geographic locations and racial groups. As an example: red hair, fair skin color or having skin that burns and never tans confer a twofold risk for developing KC.^7^ Moreover, 75% percent of BCCs occur in the head and neck region and about 20% of BCCs develop in the periocular region.^6^

KC on the eyelids has a high risk of subclinical extension and local recurrence, especially when aggressive histopathological subtypes are present.^6^ Therefore, they are considered high-risk lesions by the United States National Comprehensive Cancer Network (NCCN) classification.^8^ Periorbital skin cancer is of special concern because of the unique anatomy of the eye, eyelids, and orbit; as well as its complex functionality (e.g., eyelid function), and cosmetic relevance, given that advanced tumors of the eyelids and periorbital region can affect eye function and vision.^9^

Mohs Micrographic Surgery (MMS) is a technique that has been used successfully in the treatment of malignant skin tumors due to the complete microscopic visualization of the lateral and deep margins.^4^, ^10^ This differs from traditional methods of histopathological examination where only a portion of the margin is examined using vertical cuts, known as the “bread-loaf” technique.^5^ Studies have demonstrated that MMS has a 98%‒100% cure rate at 5-year follow-up; while using the traditional “bread-loaf” techniques, recurrence rates tend to be higher.^5^ The aim of this study is to describe the clinical-pathological features of periocular KC treated with MMS in a large Tertiary Care Center in Latin America. Secondary objectives were to evaluate variables that predict complex reconstruction.

## Methods

This study was approved by the local Institutional Review Board (ID 210818013). This study comprised a retrospective search of patients with periocular KC, that underwent MMS between January 2017 to November 2022 at a single tertiary care center. All patients who had MMS during this period were identified using electronic database records.

The authors included patients who underwent MMS with the diagnosis of periocular KC (defined as those involving the medial canthus, lateral canthus, upper eyelid, lower eyelid, and eyebrows). Tumors with histological confirmation or with a high index of suspicion (based on clinical and dermoscopic criteria) for KC (either BCC or SCC) were included. Patients with other diagnosis (e.g., melanoma) and lesions on other facial or body sites were excluded. Demographic and tumor data was recorded, such as age, gender, tumor site, recurrence status, prior treatment, histopathological subtype, pre-operative size (length and width, in mm), number of MMS stages, post-operative size of the defect (length and width, in mm), reconstruction type, and final histology. Lesion and defect size were used to calculate the surface area using the formula: (length/2 × width/2) × π, as previously described.^11^ Tumor recurrence was defined as the development of a new lesion on the same treated area in a patient who underwent any prior treatment with complete removal intention and a biopsy that demonstrated the presence of KC.

All MMS surgeries were performed by any of 5 Mohs surgeons, all of them with more than 5 years of experience in this procedure. A standard MMS fresh frozen technique was performed, with an initial excision of the tumor and subsequent tissue mapping with colored markings for orientation. The excised tumor was then analyzed microscopically by an expert ocular pathologist. MMS maps were drawn for each tumor. Subsequent excisions (if needed) were guided by the frozen section’s slides. This process was repeated until clear surgical margins were achieved. Reconstruction techniques were classified as primary closure, flap, graft, or second intention healing, performed either by dermatologists or oculoplastic surgeons. All patients subsequently underwent follow-up with any of the 5 Mohs surgeons. Recurrence after MMS was also defined as the appearance of any tumor in or around the MMS scar.

### Statistical analysis

Data were analyzed using SPSS version 25 (Armonk, NY: IBMCorp.). After verifying the non-normal distribution of the variables, the Yates Chi-Squared test and the *t*-test (two-tailed with unequal variance) tests were performed. Statistical significance was regarded as p-value < 0.05. The number of stages was categorized to 1 or 2 or more (≥2) for statistical analyses. “Complex reconstruction” was defined as a flap or graft. Analysis was performed by the intention to treat. For the determination of a cutoff point in which the initial tumor size (mm) and the initial tumor area (mm^2^) predict the type of repair, the authors tested different cut-off points on the numerical variable, and the cut-off point that yielded the highest value of the Chi-Square test (lowest p-value) was selected.

## Results

**Figure 1:**
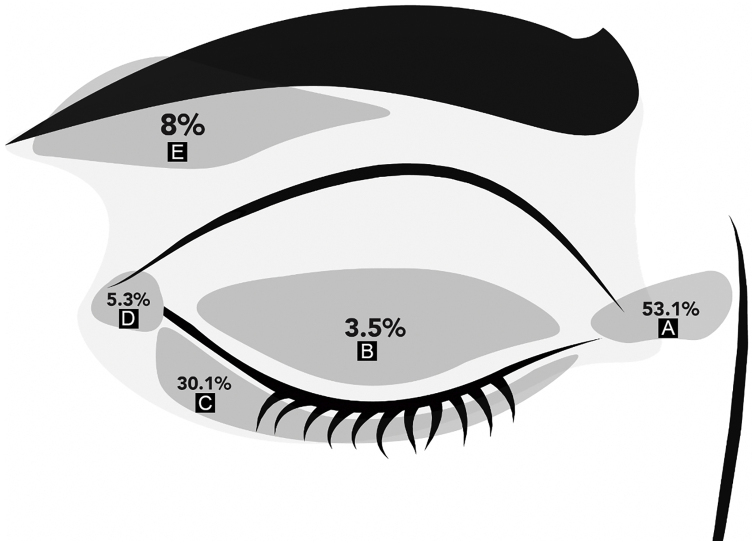
Periocular tumors location. (A) Medial canthus. (B) Upper eyelid. (C) Lower eyelid. (D) Lateral canthus. (E) Eyebrow.

There were 113 periocular MMS performed in the study period. There were 58 women and 55 men with a mean age of 59 ± 13 years (range 27‒83). KC was located on the medial canthus in 60 patients (53.1%), 34 on the lower eyelids (30.1%), and 4 on the upper eyelids (3.5%). Fig. 1 shows the distribution of KC locations on the periocular skin. Regarding the laterality of the tumor: 59 (52%) lesions were located on the right periocular region, 48 (43%) on the left, and 6 (5%) had no laterality described.

In all, 109 lesions were BCCs (105 primary and 4 recurrent); there were 67 nodular (59%), 20 micronodular (18%), 12 morpheaform (10.6%), 6 superficial (5.3%), 2 infiltrative (1.8%), and 2 subtypes were not described (Table 1). There were 4 recurrent BCCs, of which two were nodular, 1 was morpheaform, and 1 was micronodular. These 4 recurrent BCC cases had a prior tumor excision (not MMS) performed elsewhere.

**Table 1:** Patient and tumor characteristics.

Sex, n (%)	
Male	55 (49)
Female	58 (51)
Age (mean + SD) years	58.5 + 13
Tumor Type, n (%)	
BCC	109(96)
Superficial	6 (5.3)
Nodular	67 (59.3)
Micronodular	20 (17.7)
Morpheaform	12 (10.6)
Infiltrative	2 (1.8)
Unknown subtype	2 (1.8)
SCC	4 (3.5)
Tumor laterality, n (%)	
Right	59 (52.2)
Left	48 (42.5)
No laterality	6 (5.3)
Prior treatment (recurrent tumors), n (%)	5 (4)
	−4 BCC
	−1 SCC
Complications after MMS (e.g., epiphora, ectropion, etc.), n (%)	1 (0.8)

BCC, Basal Cell Carcinoma; SCC, Squamous Cell Carcinoma; SD, Standard Desviation; MMS, Mohs Micrographic Surgery.

Only 4 lesions corresponded to SCC (3.5%), all were well-differentiated and only one was a recurrent SCC.

### Mohs micrographic surgery details

**Figure 2:**
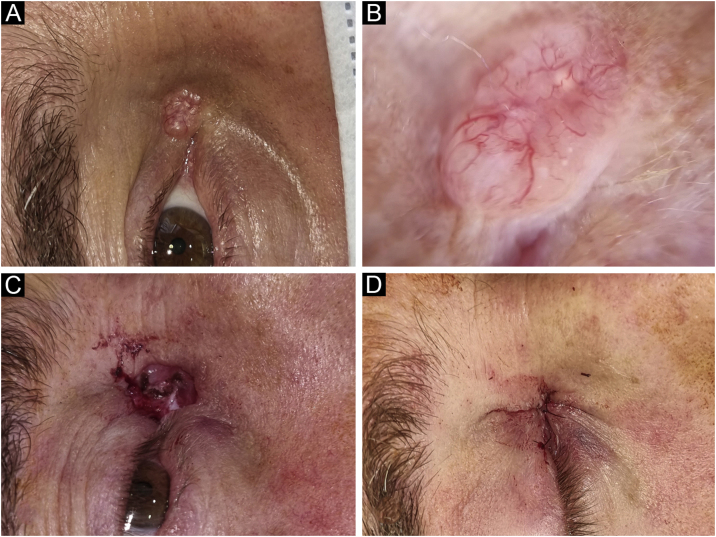
Basal Cell Carcinoma (BCC), nodular subtype. Reconstruction with primary closure. (A) Tumor located on the medial canthus on an adult Male. (B) Dermoscopy showed in-focus arborizing telangiectasia, characteristic of BCC. (C) Mohs micrographic surgery was performed achieving tumor-free margins in 1 stage. (D) Primary closure was used for repair, without tension.

**Figure 3:**
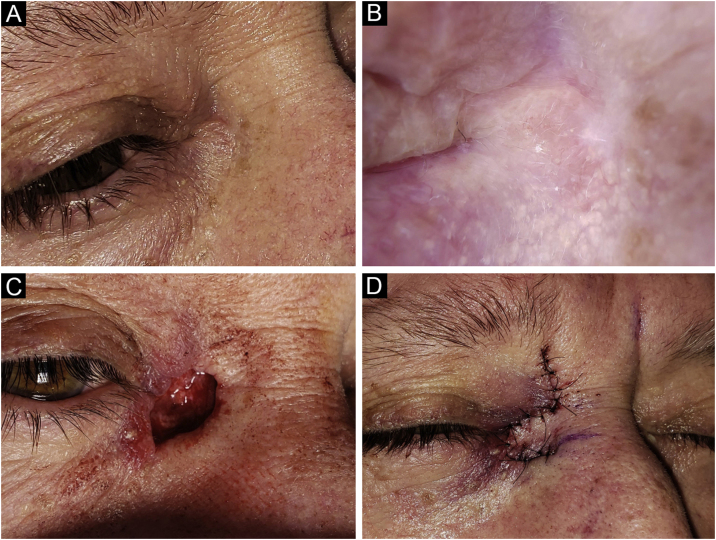
Basal Cell Carcinoma (BCC), micronodular subtype. Reconstruction with a transposition flap. (A) Tumor was located on the medial canthus on an adult female. (B) Dermoscopy showed in-focus arborizing short-fine telangiectasia and multiple aggregated yellow-white globules, characteristic of BCC. (C) Mohs micrographic surgery was performed achieving tumor-free margins in 2 stages, creating a deep canthal defect. (D) A rhomboidal flap was used to close the defect, without tension.

**Figure 4:**
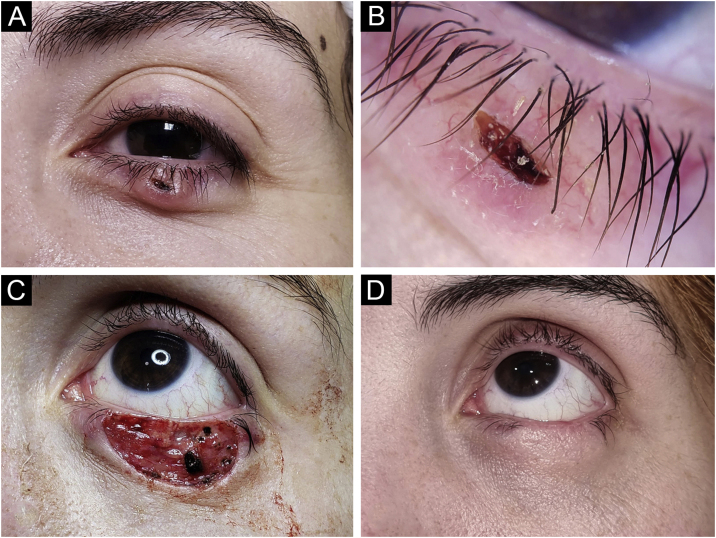
Basal Cell Carcinoma (BCC), nodular subtype. Reconstruction with a graft. (A) Tumor located on the lower eyelid in a young Female. (B) Dermoscopy showed in-focus arborizing telangiectasia and central ulceration, characteristic of BCC. (C) Mohs micrographic surgery was performed achieving tumor-free margins in 2 stages. Note the tarsal defect in the central deep margin. (D) Reconstruction with full thickness skin graft obtained from the upper eyelid was performed with excellent cosmetic results.

The mean initial tumor size was 9.21 ± 5.4 mm in the major axis and 6.76 ± 3.7 mm in the minor axis. The mean number of stages of MMS was 1.5 ± 0.7. Fifty-seven percent of the lesions were cleared in the first stage, 39% were cleared in the second stage, and 4% required 3 or more stages (Table 2). Initial tumor size (largest diameter), tumor subtypes (i.e., morpheaform, infiltrative, micronodular), age, and gender were not associated with the number of stages (p = 0.83, 0.8, 0.25, and 0.9, respectively). The mean initial tumor area was 55 ± 79 mm^2^. The initial area was not associated with the number of MMS stages (p = 0.952). Figs. 2‒4 show examples of MMS patients.

**Table 2:** Mohs Micrographic Surgery Features.

***Initial tumor size (mean + SD)***	
Major axis	9.21 ± 5.4 mm
Minor axis	6.76 ± 3.7 mm
Area (mm²)	55 + 79 mm²
***Final defect size (mean + SD)***	
Major axis	14.6 ± 8 mm
Minor axis	11 ± 6 mm
Area (mm^2^)	80 ± 104 mm^2^
***Mohs micrographic surgery details***	
*Number of stages (n = 113)*	
Mean + SD	1.5 ± 0.7
1 stage, n (%)	64 (57)
2 stages, n (%)	44 (39)
3 stages, n (%)	2 (1.5)
4 stages, n (%)	2 (1.5)
5 or more stages, n (%)	1 (1)
***Reconstruction type, n (%)***	
Primary	60 (53.1)
Flap	49 (43.3)
Graft	2 (1.8)
Secondary intention healing	1 (0.9)
Unknown	1 (0.9)
Upstaging, n (%)	4 (3.5)

### Reconstruction characteristics and predictors

The final defect size was 14.6 ± 8 mm in the major axis and 11 ± 6 mm in the minor axis. Reconstruction was performed using primary closure (53.1%), flap (43.3%), graft (1.8%), and secondary intention healing (0.9%). In 1 patient (0.9%) there was no information on reconstruction type. Larger tumor diameter (mean largest size 12.1 vs. 6.91 mm; p < 0.001) was associated with a complex repair (i.e., flap or graft). Additionally, larger defect size (19 vs. 11.2 mm, p = 0.002) and initial area (90 ± 110 mm^2^ vs. 28 ± 24 mm^2^), were also associated with a complex repair (p < 0001). When performing approximate cut-off points for complex repair, an initial tumor size ≥ 8.5 mm in the major axis and an initial tumor area ≥43.5 mm^2^ were associated with a complex repair (p < 0.001 for both).

There were 4 cases that had upstaging during MMS (i.e., a more aggressive subtype found than in the initial biopsy) in BCC tumors. They all had an initial biopsy of nodular or superficial BCC and a morpheaform BCC subtype was found on the lateral/deep margins of the 4 cases. There were no differences between biopsy type (i.e., shave vs punch) and upstaging (p = 0.38). There was one (0.8%) postoperative complication (epiphora and ectropion after MMS on the lower eyelid) and no local recurrences to date (mean follow-up 23 ± 17 months; range 2‒71 months).

## Discussion

In this retrospective study, including 113 patients with periocular KC, the authors have described the clinical-pathological characteristics of MMS-treated tumors over a 5-year period in a large academic center in Latin America. No clinical and histologic variables (age, sex, initial tumor size, and aggressive tumor subtypes) were associated with the number of stages. Larger tumor diameter (12 vs. 6.98 mm; p < 0.001) as well as larger tumor area were associated with a complex repair (i.e., flap or graft) with specific cutoff points (> 8.5 mm and > 43.5 mm^2^) predicting the need for complex repair. The present results confirm the efficacy (no recurrences) and safety (< 1% complications) of MMS in the setting of periocular KC tumors, a complex cosmetic and functional anatomical location. Since roughly 90% of cases would have been cleared with 4 mm margins (2 stages), the present findings also confirm that there might be considerable subclinical extension beyond guidelines-recommended margins in 10% of periocular cases, justifying the need for margin-controlled surgery, such as MMS.

Only a few similar descriptive studies have been reported worldwide and the present results are consistent with prior reports; however, few studies come from Latin America.^10^, ^11^, ^12^, ^13^, ^14^, ^15^, ^16^, ^17^, ^18^ In this study, the most frequent periocular KC was BCC, the nodular subtype, and the most frequent location was the medial canthus. This anatomical location is of paramount importance since tumors located on the medial canthus have a greater capacity to invade locally and destroy complex functional structures in or around the eye.^19^, ^20^ Monheit et al. report a 10-year retrospective study of 289 periocular tumors. BCCs comprised 83% of the lesions and most were nodular (76%), similar to the present findings.^9^ Scofield et al. reported 42 patients with periocular skin cancer, 34 were BCC and 8 were SCC.^12^ In Halloran et al. series, 690 patients with periocular tumors were included, BCC was the most commonly excised tumor (85.4%).^11^

In contrast to the present series, in all of these studies, BCC was most commonly located on the lower eyelid, followed by the medial canthus.^17^, ^18^, ^21^ This might be explained by local variations in sun exposure patterns (e.g., winter/summer variations, latitude), tumorigenesis, as well as intrinsic and ethnic differences in Latin America. Also, the prevalence of SCC tumors in this series was much lower compared to the literature, it could also be explained by some of these factors.^3^, ^12^, ^20^, ^22^ The mean number of stages in MMS was similar to the O’Halloran et al series (the mean number of stages was 1.5 for cases, in Australia); but it was lower compared to Scofield et al. series in which the average number of stages required during MMS to excise the cancer was 2.2 ± 1.4 stages with a range of 1–7 stages (United States).^11^, ^12^ Also, it was lower compared with Sanchez et al. series in which the average number of stages was 2.3 ± 0.9.^16^ Furthermore, in the Scofield et al. series, the prevalence of SCC was higher than in the present series (19% vs. 3.5%) and in Sanchez et al. series the proportion of aggressive subtypes was higher than in our series (50% vs. 30%).^12^, ^16^ These variables might explain the different number of stages in comparison to our study and might also reflect local variations vs. local referral/selection bias.

To the best of our knowledge, this study is the first to establish an association between the size of the initial tumor and reconstruction type. Also, the area and length of the initial major axis were associated with a complex reconstruction type which has not been previously reported. More specifically, tumors larger than 8.5 mm were associated with complex repairs. This information might be used in the preoperative setting to set patients' expectations and perform appropriate counseling (see below). In O’Halloran et al series, the mean preoperative lesion size for cases repaired by both Mohs surgeons and oculoplastic surgeons was 0.5 cm^2^, similar to the present study; however, they did not establish an association between the initial major axis and reconstruction type or a number of MMS stages.^11^ It seems reasonable that larger tumors might need more complex reconstructions as more tissue is removed. From the oculoplastic literature, it is known that full-thickness margin-involving upper or lower eyelid defects that are 35%‒50% of the length of the eyelid typically cannot be closed with primary reconstruction alone due to significant tension. In these situations, different types of flaps or grafts are excellent alternatives for periocular reconstruction.^23^ The simple size measure is a more encompassing and easy-to-apply rule to predict complex closures.

Determining an approximate Mohs defect size based on the preoperative lesion size is relevant in MMS reconstruction surgical cases, especially with regard to preoperative surgical planning/discussion and managing patient expectations. Predictive machine learning models have been developed to estimate the number of stages as well as the complexity of reconstruction.^18^, ^24^ Tan et al. presented a predictive model on the complexity of reconstructive surgery after periocular BCC excision using 3 variables that strongly predicted greater complexity: Large tumor size, delay to surgery, and risk stratification at first specialist appointment.^25^ These variables could provide a useful and simple triaging system to predict case complexity and prioritization of treatment of KC.^25^ However, when tested by the present group with local data, predictions were not as good as expected.^24^ Larger diverse cohorts and MMS performed in different settings are needed in order to properly assess and validate such machine learning models. Based on the results from the present study, one can inform the patient that tumors larger than 8.5 mm might need complex reconstruction.

Many Patient-Reported Outcome Measures (PROMs) have been used to study quality of life (QoL) in the skin cancer population, but none are specifically designed and validated for periocular KC. Gathering information from this specific anatomical location is relevant for creating and validating PROMs.^26^ PROMS could aid in identifying patients at risk for poor outcomes and poor satisfaction, serving as a guide in the selection of the most appropriate treatment.^27^, ^28^ Given the aging population and increased prevalence of KC, PROM emerges as an important tool for clinicians to assess outcomes in future interventional studies aimed at minimizing morbidity and maximizing QoL for these skin cancer patients. The advent of non-invasive imaging such as reflectance confocal microscopy might shed light on removing the ‘unknown factor’ prior to MMS being also promising tool for diagnosis and treatment decisions of periocular KC.^29^, ^30^, ^31^, ^32^, ^33^

Finally, periocular KC is complex and requires the participation of multiple medical specialties such as dermatology, oculoplastic surgeons, plastic surgeons, and pathologists, among many others. Multidisciplinary team meetings, also known as Tumor Board Conferences (TBCs), provide an opportunity to discuss the full spectrum of diagnostic, therapeutic, and social issues related to individual patients and to develop a coordinated plan for patients with complex skin cancer. These multidisciplinary meetings are often a cornerstone of treatment at leading cancer centers, having a significant impact on patient care. However, even if a tumor board is the ideal setting to discuss complex clinical cases, no guidelines are currently available on which specialists should be included in ocular oncology TBCs.^34^, ^35^ The authors encourage discussion of select periocular KC cases at TBC whenever feasible.

### Limitations

Retrospective design and the relatively low number of patients included, especially in the SCC group, which was relatively small and underrepresented, limits some of the conclusions for SCC. Possible selection bias, as larger or more complex cases, may have been referred to oculoplastic surgeons directly; nevertheless, the average defect size and involvement of posterior laminar structures were similar to those in previously published studies from oculoplastic surgeons only. The generalizability of this study is also limited because all participating MMS surgeons were academic, fellowship-trained Mohs surgeons, and the tissue was evaluated by an ocular pathologist. Finally, despite no recurrences to date, the present results should be interpreted with caution, since 60% of KCs can recur between 5 to 10 years after surgery.^36^ Therefore, longer follow-up is needed.

## Conclusion

MMS is particularly well-suited for KC of the periocular area because it allows for the highest cure rate and lower recurrence while sparing the maximal amount of normal surrounding skin and tissue. The present study establishes an association between the length of the initial major axis and the number of stages. An initial tumor size ≥8.5 mm in the major axis and an initial tumor area ≥43.5 mm^2^ were associated with a complex repair. Timely diagnosis and management of periocular malignancies is essential because of their proximity and potential to invade functional eye structures such as the orbit, eyelid, lacrimal duct, sinuses, and brain. The present results confirm the MMS success rates for primary periocular KC are excellent in Latin American patients.

## Financial support

Partial funding was obtained from La Fondation La Roche Possay Research Awards. The funding source had no role in the study.

## Authors’ contributions

Dominga Peirano: Study concept and design; collection, analysis, and interpretation; statistical analysis; writing of the manuscript; critical review of the literature; final approval of the final version of the manuscript.

Sebastián Vargas: Collection, analysis, and interpretation; statistical analysis; writing of the manuscript; critical review of the literature; approval of the final version of the manuscript.

Leonel Hidalgo: Collection, analysis, and interpretation; writing of the manuscript; critical review of the literature; approval of the final version of the manuscript.

Francisca Donoso: Collection, analysis, and interpretation; writing of the manuscript; critical review of the literature; final approval of the final version of the manuscript.

Eugenia Albuseme: Study concept and design; collection, analysis, and interpretation; writing of the manuscript; critical review of the literature; approval of the final version of the manuscript.

Felipe Sanhueza: Study concept and design; collection, analysis, and interpretation; writing of the manuscript; critical review of the literature; approval of the final version of the manuscript.

Consuelo Cárdenas: Study concept and design; collection, analysis, and interpretation; writing of the manuscript; critical review of the literature; approval of the final version of the manuscript.

Katherine Droppelmann: Study concept and design; collection, analysis, and interpretation; statistical analysis; writing of the manuscript; critical review of the literature; approval of the final version of the manuscript.

Juan Camilo Castro: Study concept and design; collection, analysis, and interpretation; writing of the manuscript; critical review of the literature; approval of the final version of the manuscript.

Pablo Uribe: Study concept and design; collection, analysis, and interpretation; writing of the manuscript; critical review of the literature; approval of the final version of the manuscript.

Pablo Zoroquiain: Study concept and design; collection, analysis, and interpretation; writing of the manuscript; critical review of the literature; final approval of the final version of the manuscript.

Cristian Navarrete-Dechent: Study concept and design; collection, analysis, and interpretation; statistical analysis; writing of the manuscript; critical review of the literature; approval of the final version of the manuscript.

## Conflicts of interest

None declared.
